# Improving the diagnosis of common parotid tumors via the combination of CT image biomarkers and clinical parameters

**DOI:** 10.1186/s12880-020-00442-x

**Published:** 2020-04-15

**Authors:** Dan Zhang, Xiaojiao Li, Liang Lv, Jiayi Yu, Chao Yang, Hua Xiong, Ruikun Liao, Bi Zhou, Xianlong Huang, Xiaoshuang Liu, Zhuoyue Tang

**Affiliations:** 1 Department of Radiology, Chongqing General Hospital, University of Chinese Academy of Sciences, No.104 Pipashan Main St, Yuzhong District, Chongqing, 400014 China; 2 Molecular and Functional Imaging Laboratory, Chongqing General Hospital, University of Chinese Academy of Sciences, Chongqing, 400014 China; 3 Department of Radiology, The First Affiliated Hospital of Chongqing Medical University, Chongqing, 400016 China

**Keywords:** Parotid tumors, Image biomarkers, Clinical parameters

## Abstract

**Background:**

Our study aims to develop and validate diagnostic models of the common parotid tumors based on whole-volume CT textural image biomarkers (IBMs) in combination with clinical parameters at a single institution.

**Methods:**

The study cohort was composed of 51 pleomorphic adenoma (PA) patients and 42 Warthin tumor (WT) patients. Clinical parameters and conventional image features were scored retrospectively and textural IBMs were extracted from CT images of arterial phase. Independent-samples t test or Chi-square test was used for evaluating the significance of the difference among clinical parameters, conventional CT image features, and textural IBMs. The diagnostic performance of univariate model and multivariate model was evaluated via receiver operating characteristic (ROC) curve and area under ROC curve (AUC).

**Results:**

Significant differences were found in clinical parameters (age, gender, disease duration, smoking), conventional image features (site, maximum diameter, time-density curve, peripheral vessels sign) and textural IBMs (mean, uniformity, energy, entropy) between PA group and WT group (*P*<0.05). ROC analysis showed that clinical parameter (age) and quantitative textural IBMs (mean, energy, entropy) were able to categorize the patients into PA group and WT group, with the AUC of 0.784, 0.902, 0.910, 0.805, respectively. When IBMs were added in clinical model, the multivariate models including age-mean and age-energy performed significantly better than the univariate models with the improved AUC of 0.940, 0.944, respectively (*P*<0.001).

**Conclusions:**

Both clinical parameter and CT textural IBMs can be used for the preoperative, noninvasive diagnosis of parotid PA and WT. The diagnostic performance of textural IBM model was obviously better than that of clinical model and conventional image model in this study. While the multivariate model consisted of clinical parameter and textural IBM had the optimal diagnostic performance, which would contribute to the better selection of individualized surgery program.

## Background

Parotid gland neoplasms are the most common type of salivary gland neoplasms, with 75–80% being benign [1]. Pleomorphic adenoma (PA) and Warthin tumor (WT) are the two most common types of the parotid gland tumors, and the incidence of WT has been gradually increasing nowadays [1, 2]. Although both of them are benign tumors, the biological characteristics and surgery programme are completely different. PA may develop into malignant tumor as a result of a delayed surgery, and postoperative recurrence of PA is very common. Yet, WT grows slowly, rarely recurs and malignant transformation seldom occurs [3, 4]. At present, partial superficial parotidectomy (PSP) is the most common surgical procedure for PA and extracapsular dissection (ECD) for WT, which is in line with the current trend of minimising surgical dissection. Therefore, the risk of short-term and long-term complications might be decreased [5]. ECD is a safe and time-efficient surgical approach, offering earlier recovery and better preservation of salivary function compared to PSP. Meanwhile, ECD should be considered as a surgical approach for parotid tumors, especially those in the parotid tail, such as WT [6]. Yet, ECD is not quite suitable to be applied to PA, for it will increase the postoperative recurrence rate compared to PSP.

Preoperative knowledge of the pathological type of tumors would be of great importance in consideration of optimizing the individualized operative program and help to inform preoperative patient counselling. In routine clinical practice, common factors such as age, performance status, tumor size, site, and the like, are used to guide treatment decision-making. Nevertheless, patients with similar factors above may have different outcome. Neither these clinical factors nor conventional image features are sufficient for identifying patients that will benefit most from specific surgical strategies. Thus, more detailed information that reflect the characteristics of the whole tumor are needed to improve the diagnostic accuracy.

Some recent reports have indicated that IBMs of tumors are prominently related to the pathology and prognosis [7, 8]. IBMs can be extracted from various medical images and provide quantitative information with regard to shape, intensity and texture features of the region of interest (ROI) [9]. There is plenty of quantitative information on medical images, far beyond our current understanding. As a result, there is an increasing interest in the assessment of tumors on medical images by advanced software in order to derive additional, clinically relevant information, namely texture analysis. Texture analysis has been used to predict several kinds of clinical issues, such as tumor heterogeneity, patient prognosis, and response to therapy [10–12]. In this study, texture features were extracted from CT images and incorporated into the diagnostic model for histological classification of the two most common benign parotid tumors, WT and PA.

Many IBMs of tumors are significantly related to the outcome, but it is still unclear to what extent the addition of IBMs improves the diagnostic performance of models consisted of clinical parameters and conventional image features. Moreover, there is a relative paucity of literature as for the combined model. The aim of this study was to test whether the diagnostic performance of prediction models could be improved by the addition of IBMs compared to models based on solely clinical parameters or conventional images for WT and PA.

## Methods

### Patient selection

This retrospective study was approved by the local ethics review board. The informed consent was waived for this single-institution study due to its retrospective nature. All data of patients were used confidentially and anonymously. The research involved no more than minimal risk to the patients. Meanwhile, the waiver did not adversely affect the rights and welfare of the patients. Clinical and image data of all patients were obtained through medical record system and follow-up. Between January 2016 and May 2017, patients with PA or WT who underwent surgery were eligible and identified from the institution’s database.

**Fig. 1:**
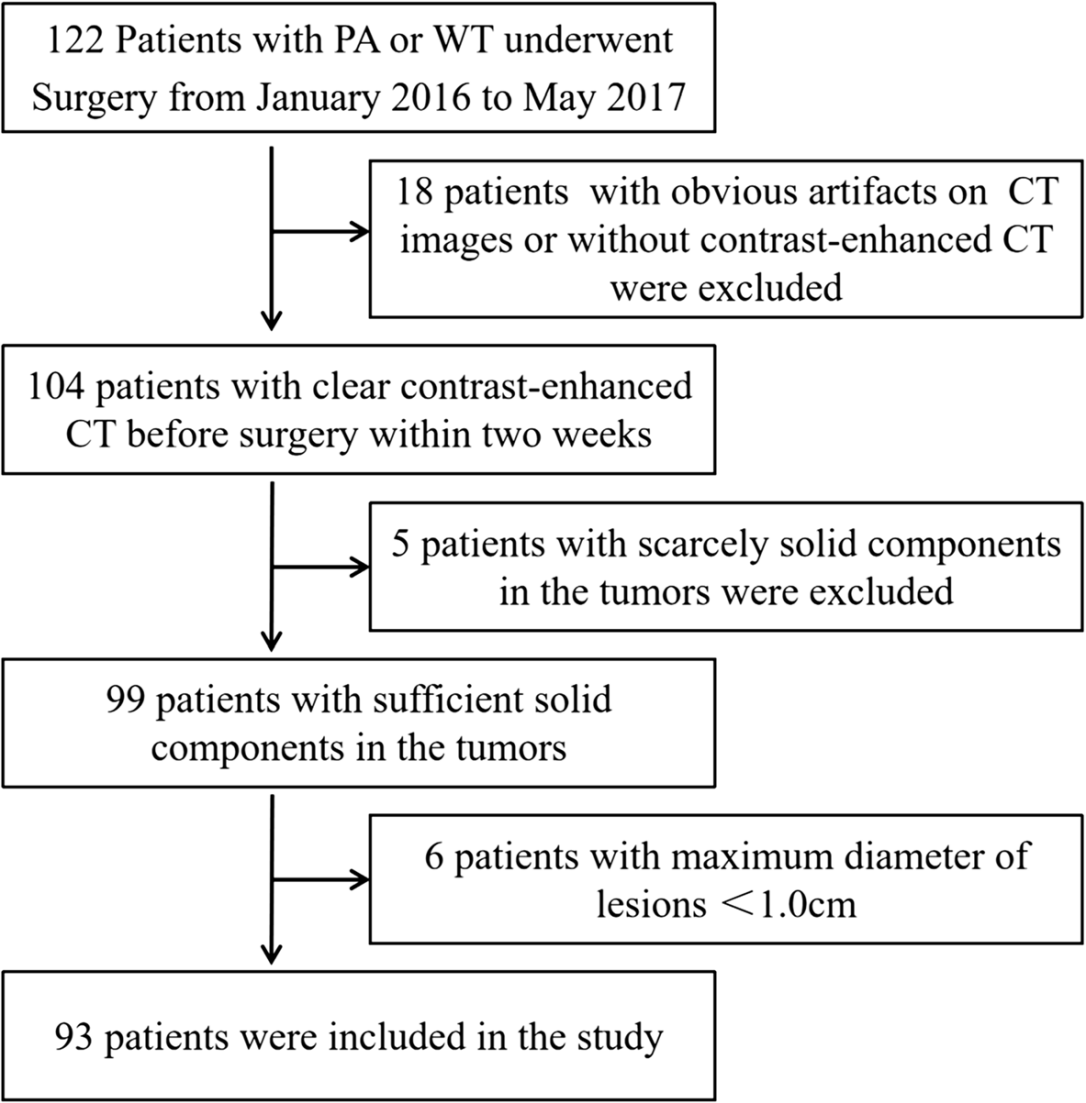
Schematic shows recruitment pathway of patients for this study

The inclusion criteria were as follows: (1) confirmed PA or WT with postoperative pathological diagnosis; (2) contrast-enhanced CT images of neck containing parotid gland obtained within 2 weeks prior to surgery; and (3) maximum diameter of lesions ≥1.0 cm. The exclusion criteria were as follows: (1) CT images with obvious artifacts, such as false teeth artifacts, motion artifacts, etc.; and (2) lesions with scarcely solid components which are difficult for texture analysis. As a result, a total of 122 patients were identified, and 29 patients were excluded (Fig. 1). The final study population comprised 93 patients.

### Image acquisition

CT scans were performed using 64/128-multidetector scanners (LightSpeed VCT; GE Healthcare, Waukesha, WI, USA) and the parameters were as follows: tube current, 150 mA; tube voltage, 120 kVp; section thickness, 5 mm; section interval, 5 mm.

The scanning ranges from the base of the skull to the entrance to the thorax. The enhanced images were obtained after intravenous injection of 80–100 mL of nonionic contrast material (320 mg/ mL; Iopamidol, Shanghai Bracco Sine Pharmaceutical Co., Ltd., Shanghai, China) at an injection rate of 3.0 mL/s, followed by a 50 mL saline chaser. The contrast-enhanced CT images were obtained at 35 and 120 s after contrast material injection in arterial phase and balanced phase, respectively.

### Clinical parameters

All clinical parameters including age, gender, disease duration, and smoking status were collected from medical record system.

### Conventional CT image features

All conventional CT image features were derived from the original CT image data, including tumor site (in the parotid tail or not), maximum diameter, time-density curve (washout type or not), and peripheral vessels sign, which defined as increased tortuous vascular shadows clinging to the edge of the lesion.

### CT textural image biomarkers

**Fig. 2:**
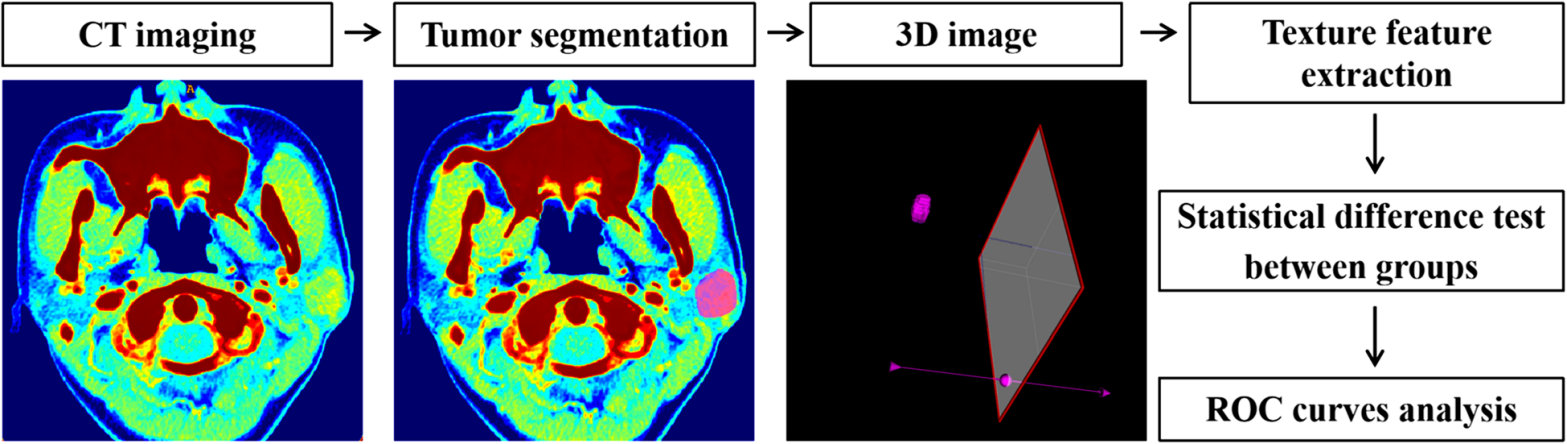
Flowchart illustrating the texture analysis in this study

CT images of arterial phase of all patients were stored in Digital Imaging and Communications in Medicine (DICOM) format and uploaded to ITK-SNAP software for three-dimensional manual segmentation of the region of interest (ROI). An ROI was manually drawn to cover the tumor as large as possible, keeping a distance of 1 mm from the boundary and carefully avoiding the retromandibular vein and too much parotid parenchyma into the lesion, which may lead to a misunderstanding of the internal structure of the tumor and affect the accuracy of texture analysis. The ROI of each case was manually drawn by a head and neck radiologist who did not have any knowledge about the clinical information of patients, and then the segmentation was checked by a senior radiologist. Areas of tumor heterogeneity, including cystic change or necrosis, were not excluded, for the information captured with texture analysis could potentially contribute to tumor discrimination and classification. An in-house software, Matlab2017b (Mathworks, Natick, MA, USA), was used to extract the texture parameters automatically. Six frequently-used texture parameters obtained from the gray-level histogram were included in the study, namely uniformity (a measure of the sum of the squares of each intensity value), mean (the average gray level intensity within the ROI), energy (a measure of the magnitude of voxel values in an image), entropy (the distribution of gray levels within the VOI), skewness (the histogram asymmetry degree around the mean), and kurtosis (a measurement of the histogram sharpness). An overview of the textural IBM extraction process and analysis is shown in Fig. 2.

### Data analysis

The data were analyzed by SPSS 22.0 software (IBM, Chicago). Kolmogorov-Smirnov test was used for intra-group normality test, and Levene test was used for intra-group variance homogeneity test. Parameters with normal distribution and homogeneity of variances were expressed as mean ± standard deviation, and independent-samples t test was adopted for data analysis. Parameters that did not satisfy normal distribution and uneven variance were expressed by median and interquartile spacing. The two-independent-samples Mann-Whitney U Test was used for data analysis. Qualitative data were presented as ratios, which were analyzed by chi-square test. As to clinical parameter (gender) - textural IBM models, a two-component diagnostic model was fitted with binary Logistic regression analysis. The diagnostic performance of each index was tested via receiver operating characteristic (ROC) analysis. Cutoff values were established by calculating the maximal Youden index (Youden index = sensitivity+specifcity-1). A *P* value was considered significant if it was less than 0.05.

### Step 1 clinical model

Potential clinical parameters that were considered for their diagnostic ability in the PA and WT datasets included age (>median vs. ≤ median), gender (female vs. male), disease duration (>median vs. ≤ median), and smoking status (yes vs. no).

### Step 2 conventional image model

Potential conventional CT image features that were considered for their diagnostic ability in the PA and WT datasets included tumor site (in the parotid tail, which can be defined as inferior 2 cm of the superficial lobe of the gland, vs. not), maximum diameter (>median vs. ≤median), time-density curve (TDC) (washout type vs. not), peripheral vessels sign, which is defined as increased tortuous vascular shadow clinging to the edge of lesion in the arterial phase (yes vs. no).

### Step 3 textural IBM model

Six texture features namely mean, uniformity, energy, entropy, skewness and kurtosis were calculated and selected. The potential textural IBMs were analyzed for their diagnostic power, and the median values (>median vs. ≤ median) in the WT and PA datasets were regarded as the threshold value in the univariate analysis.

### Step 4 combined models

According to the ROC analysis and AUC, the potential clinical parameters, conventional image features, and textural IBMs were included in the multivariate analysis to create combined models, and the optimal one was selected out.

## Results

### Step 1 clinical model

Univariate analysis showed the age in the WT group was significantly older than that in the PA group (*P* < 0.001). The disease duration in the PA group was significantly longer than that in the WT group (*P* = 0.01). Meanwhile, significant differences were found in gender and smoking status between the two groups (*P* < 0.001). All the selected clinical parameters showed significant differences between two groups, which was shown in Table 1.

**Table 1:** Baseline patient characteristics in PA goup and WT group

Characteristics	Pleomorphic adenoma (*N* = 51)	Warthin tumor (*N* = 42)	*P* value
Age^a^ (year)	43.88 ± 17.188	59.93 ± 6.194	<0.01
Gender^b^			
Male	15 (28.4%)	41 (97.6%)	<0.01
Female	36 (70.6%)	1 (2.4%)	
Smoking status^b^			
(+)	12 (23.5%)	37 (88.1%)	<0.01
(−)	39 (76.5%)	5 (11.9%)	
Disease duration^a^ (month)	27.91 ± 36.868	12.54 ± 17.105	0.023

N Number. (+) smoked, while (−) never smoked. ^a^Data: Mean ± SD. ^b^Data: No. (percentage)

*Abbreviations*: *PA* Pleomorphic adenoma, *WT* Warthin tumor

### Step 2 conventional image model

The maximum diameter of lesion in the WT group was significantly larger than that in the PA group (2.41 ± 0.534 vs. 2.02 ± 0.588, *P* = 0.003). The WT group is more inclined to be in the parotid tail compared with the PA group (78.6% vs. 19.6%, *P* < 0.001). As to the peripheral vessels sign and washout type of TDC, they were more frequently in the WT group compared with the PA group (64.3% vs. 3.9 and 88.1% vs. 3.9%, respectively, *P* < 0.001) (Table 2).

**Table 2:** Conventional CT image features in PA goup and WT group

Parameter	Pleomorphic adenoma (*N* = 51)	Warthin tumor (*N* = 42)	*P* Value
Maximum diameter^a^ (cm)	2.02 ± 0.588	2.41 ± 0.534	0.003
Site^b^ (in the tail)			
Yes	10 (19.6%)	33 (78.6%)	<0.01
No	41 (80.4%)	9 (21.4%)	
TDC^b^ (washout type)			
Yes	2 (3.9%)	37 (88.1%)	<0.01
No	49 (96.1%)	5 (11.9%)	
Peripheral vessels sign^b^			
Yes	2 (3.9%)	27 (64.3%)	<0.01
No	49 (96.1%)	15 (35.7%)	

*N* Number. ^a^Data: Mean ± SD. ^b^Data: No. (percentage)

*Abbreviations*: *PA* Pleomorphic adenoma, *WT* Warthin tumor, *TDC* Time-density curve

### Step 3 textural IBM model

The mean, energy, and entropy of WT group were significantly higher than those of PA group (all *P* < 0.001), while the uniformity of WT group was significantly lower than that of PA group (*P* < 0.001). No statistically significant differences of skewness and kurtosis were found in both groups (*P* = 0.05 and *P* = 0.151, respectively) (Table 3).

**Table 3:** Six textural IBMs based on the CT arterial phase images in PA group and WT group

Parameter	Pleomorphic adenoma (*N* = 51)	Warthin tumor (*N* = 42)	*P* Value
Uniformity^a^	0.429 ± 0.116	0.318 ± 0.077	<0.01
Entropy^a^	1.584 ± 0.367	1.983 ± 0.303	<0.01
Mean^a^	57.664 ± 18.121	91.225 ± 19.523	<0.01
Skewness^a^	−0.455 ± 0.568	− 0.678 ± 0.613	0.072
Energy^a^	1.688E7 ± 1.708E7	7.313E7 ± 5.002E7	<0.01
Kurtosis^a^	3.958 ± 1.444	4.387 ± 1.402	0.152

Abbreviations: *IBMs* Image biomarkers, *CT* Computed tomography, *PA* Pleomorphic adenoma

^a^Data: Mean ± SD

### Step 4 diagnostic performance of univariate and multivariate models

**Fig. 3:**
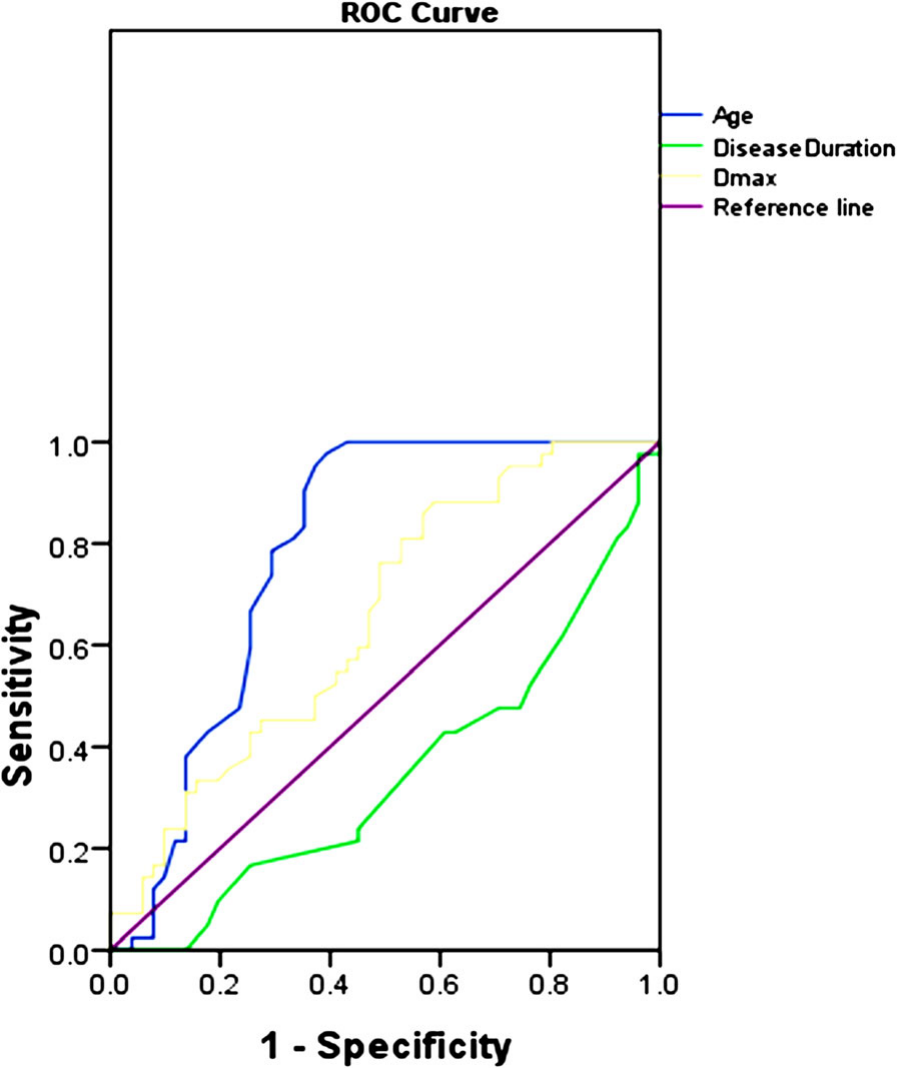
ROC curves for distinguishing WT from PA based on clinical parameters

**Fig. 4:**
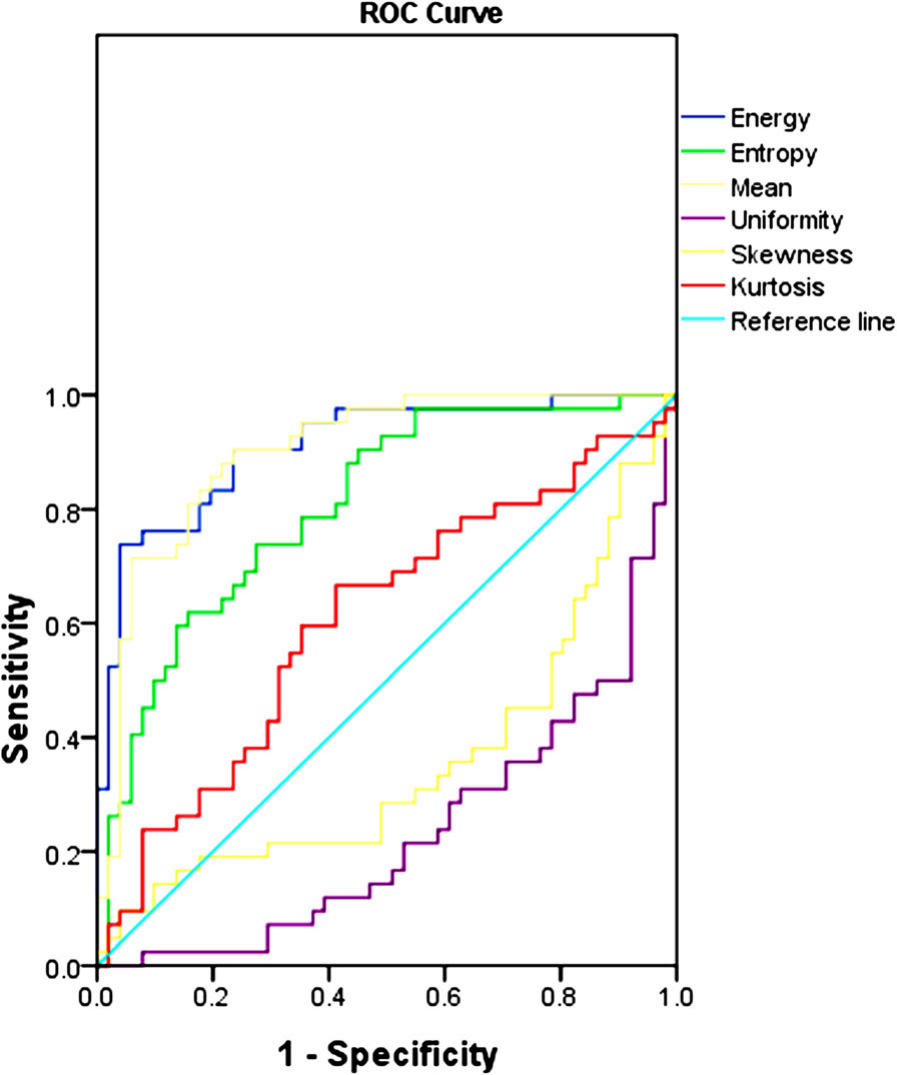
ROC curves for distinguishing WT from PA based on CT textural IBMs

**Fig. 5:**
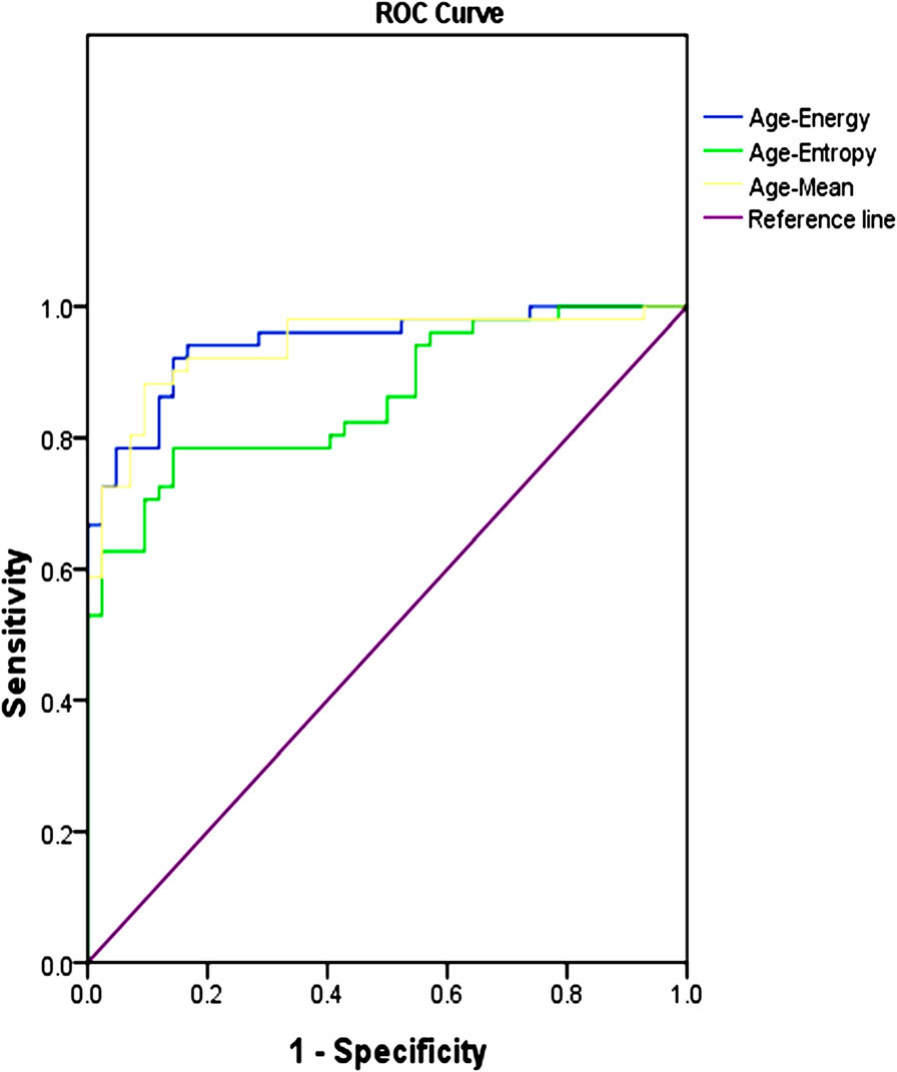
ROC curves for distinguishing WT from PA based on multivariate models, composed of clinical parameter and CT textural IBMs

Univariate analysis showed significant differences in age, gender, disease duration, smoking status, site (in the parotid tail), peripheral vessels sign, TDC (washout type), maximum diameter, mean, energy, entropy and uniformity between the WT and PA group (all *P* < 0.05). ROC analysis showed that clinical parameter (age) and textural parameters (mean, energy, entropy) performed well in differentiating the WT group from the PA group, and yielded the AUC of 0.784, 0.902, 0.910, 0.805, respectively (Figs. 3, 4, Table 4). The multivariate models were consisted of the clinical parameter and textural parameters, including age-mean, age-energy, age-entropy, with the AUC of 0.940, 0.944, 0.841, respectively. The optimal multivariate models included age-mean, age-energy, and yielded the AUC, sensitivity, and specificity of 0.940, 0.882, 0.905 and 0.944, 0.922, 0.857, respectively (*P* < 0.001) (Fig. 5, Table 5).

**Table 4:** Diagnostic performance of various indexes, including clinical parameters, conventional image features and textural IBMs

Index	AUC	*P* Value	95% CI	Cutoff value	Sensitivity	Specificity
Gender	0.171	<0.01	0.084–0.258	1.500	0.048	0.294
Age	0.784	<0.01	0.686–0.881	47.500	0.976	0.608
Disease duration	0.347	0.011	0.235–0.458	3.500	0.476	0.255
Smoking status	0.177	<0.01	0.088–0.267	1.500	0.119	0.235
Site (in the tail)	0.205	<0.01	0.109–0.301	1.500	0.214	0.196
Maximum diameter	0.652	0.012	0.541–0.762	1.83	0.881	0.412
TDC (washout type)	0.079	<0.01	0.014–0.145	1.500	0.119	0.039
Peripheral vessels sign	0.210	<0.01	0.111–0.309	1.500	0.381	0.039
Mean	0.902	<0.01	0.840–0.964	67.364	0.905	0.765
Energy	0.910	<0.01	0.851–0.970	42,558,500	0.738	0.961
Entropy	0.805	<0.01	0.717–0.893	1.767	0.738	0.725
Uniformity	0.235	<0.01	0.138–0.331	0.211	1	0.020

Abbreviations: *IBMs* Image biomarkers, *TDC* Time-density curve, *AUC* Area under ROC curve

**Table 5:** Diagnostic performance of multivariate models, consisted of clinical parameter and textural IBMs

Index	AUC	*P* Value	95% CI	Sensitivity	Specificity
Age-Mean	0.940	<0.01	0.891–0.988	0.882	0.905
Age-Energy	0.944	<0.01	0.900–0.988	0.922	0.857
Age-Entropy	0.861	<0.01	0.788–0.934	0.784	0.857

Abbreviations: *IBMs*, Image biomarkers, *AUC* Area under ROC curve

## Discussion

This study showed a detailed analysis on the different diagnostic models for PA and WT patients. We not only calculated AUC, sensitivity and specificity of univariate and multivariate model, but also analyzed the differences between the AUC values. Our results indicated that textural IBMs might be helpful to diagnose patients with parotid PA and WT. Especially, the multivariate models including clinical parameter and textural IBMs showed better diagnostic performance in this study.

Nowadays, surgeons are in great efforts to reduce the incidence of post-parotidectomy complications under the advocacy of precision medicine. PSP and ECD are the two most commonly used surgery programmes for patients with parotid tumors at present [5, 13]. ECD is often used to excise tumors located in parotid tail. It has been considerd as a minimally invasive surgery and results in fewer complications, higher efficiency, and better preservation of salivary function [6, 14, 15]. Parotid tail is defined as inferior 2 cm of the superficial lobe and lies anterolateral to sternocleidomastoid muscle [16]. The surgical plan of neoplasms located in the parotid tail may be quite different from other parotid areas, especially for WT [6]. In our study, most of WT were located in the parotid tail, while few of PA were discovered in the tail (P<0.001). Consequently, ECD is not quite suitable to be applied to PA. Furthermore, it has been proved a significantly higher rate of recurrent PA with ECD compared to PSP [17].

Parotid PA and WT always present as painless, slowly growing mass without characteristic performance in laboratory and traditional imaging examinations. It has been demonstrated that smoking and elderly male were characteristics of WT, with the ratio of male patients to female patients from 1.7:1 to 11.5:1 and the average age of patients from 56.7 years to 60 years [18, 19], which was roughly consistent with the findings of the present study. While, the PA is more prone to occur in middle-aged women (*P* < 0.001) compared to WT in this study, which was also in line with those found by the literature. This study found that disease duration of PA is longer than WT. We speculate that WT is larger in size (*P*<0.001) and more superficial in location (*P* = 0.003) compared with PA, which is beneficial to early detection.

Nevertheless, lots of overlapping features of WT and PA lead to the difficulties in the preoperative diagnosis [20]. Fine needle aspiration (FNA) regarded as the gold standard for the diagnosis of parotid tumors has several inevitable limitations and may be related to the low sensitivity and poor levels of diagnostic accuracy [21]. The false positive rates of FNA in PA and WT were reported to be 9 and 8%, respectively, and the multifarious cytomorphology of these tumors may lead to the misdiagnosis [22]. Furthermore, the heterogeneity of tumors may be underestimated from a single or limited biopsy sample [23]. Thus, pre-operation imaging examination may be a noninvasive and better approach to identify WT and PA.

At present, multi-phase contrast-enhanced CT has been applied to the preoperative examination for the patients with parotid tumors and it has been the main method for the preoperative assessment [20, 24]. As we know, washout time of contrast agent in the tumors could provide valuable pathophysiological information and be helpful for the differential diagnosis of pariod tumors [24, 25]. As to PA and WT, quick expurgation of contrast agent was unique for WT, while a delayed enhancement was unique for PA, which was consistent with the findings of the present study. But it was not always quite the case, for some WTs also present delayed enhancement and PAs present quick expurgation [20], which have been confirmed in this study. Furthermore, the delayed imaging would increase the radiation doses or reduce the temporal resolution.

Additionally, peripheral vessels sign was detected more frequently in WTs (P<0.001) compared with PAs in the arterial phase, which has not been previously reported. We speculate that WT is hypervascular lesion with abundant expanded blood capillaries according to histopathological features, which may contribute to its potential of stimulating peripheral angiogenesis.

The diagnostic efficiency of energy in all textural IBMs was the best in this cohort of parotid WT and PA patients, according to the univariate diagnostic models of this study. This was verified by the whole-volume texture analysis from CT images of arterial phase. That is to say, the textural IBM provided a stronger association with the diagnosis of WT and PA compared to clinical model and conventional image model. The textural IBMs were quantified by extracting features from the complete tumor volume in this study, which was quite different from other texture analysis of parotid disease, for the ROI was manually drawn around the tumor on its largest cross-sectional area, instead of the whole volume of the tumor [26]. In consequence, the overall tumor features were reflected by textural IBMs. In addition to some features on plain CT, contrast-enhanced CT can also reflect some heterogeneous features on tumor blood supply. Parotid gland tumors are primarily supplied by arteries, so CT images of arterial phase were selected to analyze the texture features of PA and WT in this study.

Texture features may be acquired from several kinds of image examinations, such as CT, magnetic resonance imaging (MRI), positron emission tomography (PET) and the like, without changing the acquisition protocols and additional costs for patients [27–29]. Currently, texture analysis is mainly used to evaluate the treatment effect and prognosis of lung cancer, colorectal cancer, liver cancer and so on [30–32]. But it is rarely applied to parotid gland, except for several reports focusing on the alterations of parotid morphology and secretion function induced by radiotherapy for head and neck cancers [33, 34]. However, as we know, there is a paucity of literature pertaining to the potential diagnostic value of CT textural IBMs in parotid tumors, as well as the multivariate model.

Given the different surgical management of PA and WT patients, it is hoped that gathering clinical clues and CT IMBs together could improve the ability to make treatment decisions and help predict surgical outcomes. In this study, the diagnostic performance was obviously improved by the combination of clinical parameter and textural IBMs, compared to univariate model, especially the conventional image model and clinical model. Our results suggest that with no additional imaging burden, texture analysis of preoperative routine contrast-enhanced CT imaging may give us helpful information for PA and WT patients performing different operations. As we learn more about the CT textural IBM, hopefully it will lead to more benefits for new operative scheme for patients with parotid gland tumors.

The present study had several limitations. First, the sample size was relatively small, although it was larger than previous CT texture analysis studies on PA and WT [26]. Although the sample size is relatively small, we got a strong correlation between textural IBMs and the diagnosis of PA and WT, as well as the multivariate models. The results of this research inspire investigating the relationship between broader radiomic features and parotid tumors of other pathological types in a larger sample size. Second, all patients enrolled in this study were from one institution, so a large-scale randomized controlled trial needs to be performed to validate our results. At last, there is a shortage of understanding on the potential relationship between textural IBMs and histopathology of parotid PA and WT which requires further work. Meanwhile, further research needs to be done on the repeatability of these quantifiable IBMs as part of a biomarker validation process.

## Conclusions

In conclusion, the multivariate models consisted of the clinical parameter and textural IBMs improved the preoperative diagnosis of WT and PA, and facilitated the individualized operation plan for patients. Consequently, the parotidectomy trauma may be minimized and the postoperative complication may be reduced. The CT textural IBMs are worth exploring to determine whether they can improve the clinical programme currently applied.

## Data Availability

The datasets used and analysed during the current study are available from the corresponding author on reasonable request.

## Abbreviations

IBM: Image biomarkers
PA: Pleomorphic adenoma
WT: Warthin tumor
ROC: Receiver operating characteristic
AUC: Area under receiver operating characteristic curve
PSP: Partial superficial parotidectomy
ECD: Extracapsular dissection
DICOM: Digital imaging and communications in medicine
ROI: Region of interest
VOI: volume of interest
TDC: time-density curve
FS: Frey’s syndrome
FNA: Fine needle aspiration
MRI: Magnetic resonance imaging
PET: Positron emission tomography
